# Trisomy 13 With Bilateral Congenital Anophthalmia: A Case Report

**DOI:** 10.7759/cureus.60264

**Published:** 2024-05-14

**Authors:** Hanae Bahari, Hanane Hajaj, Anass Ayyad, Sahar Messaoudi, Rim Amrani

**Affiliations:** 1 Department of Neonatology and Neonatal Resuscitation, Mohammed VI University Hospital, Oujda, MAR; 2 Mother and Child Health Laboratory, Faculty of Medicine and Pharmacy, Mohammed First University, Oujda, MAR

**Keywords:** karyotype, polydactyly, cleft lip and palate, anophthalmia, trisomy 13

## Abstract

Trisomy 13, also known as Patau syndrome, is a widely congenital anomaly syndrome characterized by microphthalmia, cleft lip, and palate, microcephaly with a sloping forehead, congenital heart disease, and polydactyly of the limbs. Patau syndrome is identified either prenatally or postnatally. Its survival rate is low, and most of the patients die even before their first year of life. The risk of trisomy 13 is higher in women of advanced maternal age. Brain and cardiovascular abnormalities are typically the primary factors contributing to the syndrome's poor prognosis. We report a case of a male newborn born at full term from a first-degree consanguineous marriage. Upon initial inspection, the patient had classic dysmorphic features, including low-set ears, a cleft lip and palate, a short neck, bilateral anophthalmia, and polydactyly of the limbs. After chromosomal analysis, the diagnosis was made, and a trisomy 13 was discovered.

## Introduction

Trisomy 13, a chromosomal aberration first described by Patau in 1960, exhibits an incidence rate of approximately one case per 10,000 births, a statistic significantly influenced by maternal age [1]. Patau syndrome is characterized by a polymalformative syndrome that presents a spectrum of abnormalities affecting various systems, including the cerebro-meningeal, cardiovascular, digestive, urogenital, limb, and trunk systems. Congenital anophthalmia is the consequence of a lack of development or regression of the primary optic vesicle in utero. This anomaly is considered rare; multiple studies have reported different incidence rates: approximately 21.34 per 100,000 newborns in a Spanish study and 10 per 100,000 births in a UK study by Busby et al. [2,3]. The prevalence of anophthalmia does not appear to be influenced by race or gender. Anophthalmia may be unilateral or, rarely, bilateral, isolated, or linked to other deformities. Many etiologies, including infections, toxic substances, chromosomal abnormalities, and gene mutations, are frequently found. For some infants, receiving appropriate surgical interventions and medical treatments at the right time has been proven to enhance their chances of survival. Introducing palliative care early on is also vital, as it helps alleviate physical and emotional suffering for both the infant and their family [4].

Trisomy 13 is a rare and fatal chromosomal mutation that typically results in unilateral or bilateral microphthalmia and, infrequently, anophthalmia [1].

In this paper, we present a new trisomy 13 case revealed with bilateral anophthalmia and multiple congenital anomalies.

## Case presentation

**Figure 1:**
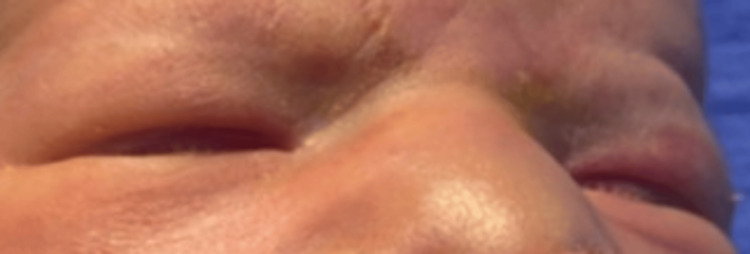
Bilateral anophthalmia

**Figure 2:**
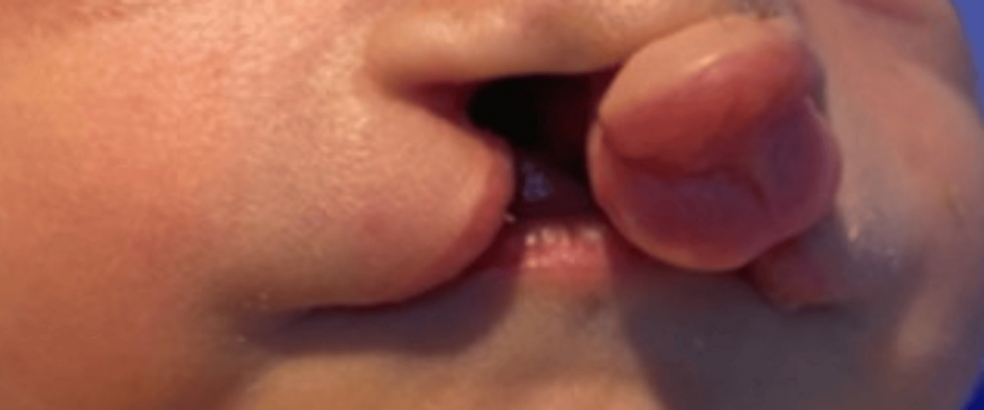
Median cleft lip and palate

**Figure 3:**
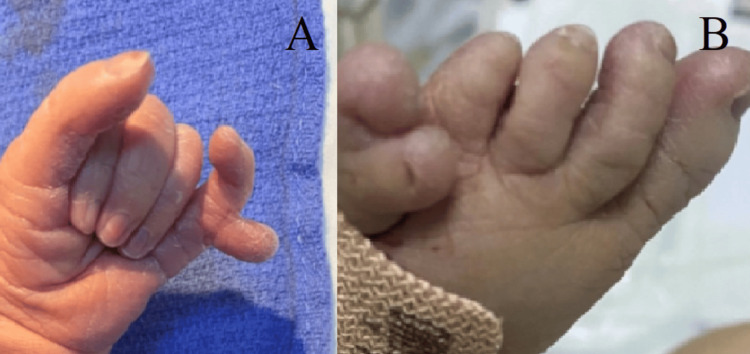
Polydactyly of the limbs; (A) polydactyly of the left hand, (B) polydactyly of the left foot

We present the case of a male newborn, the third child in the family with a positive history of first-degree consanguinity and a poorly followed pregnancy estimated at 38 weeks of gestation, to a 40-year-old mother via an unremarkable vaginal delivery, with an APGAR (activity, pulse, grimace, appearance, and respiration) score of 7/10. During her pregnancy, the mother had never used medication before. The weight, length, and head circumference measurements at birth are 3.1 kg, 49 cm, and 35 cm, respectively. On initial examination, it showed dysmorphic features like bilateral anophthalmia (Figure 1), low-set ears, a short neck, a median cleft lip and palate (Figure 2), as well as polydactyly of the limbs (Figure 3).

**Figure 4:**
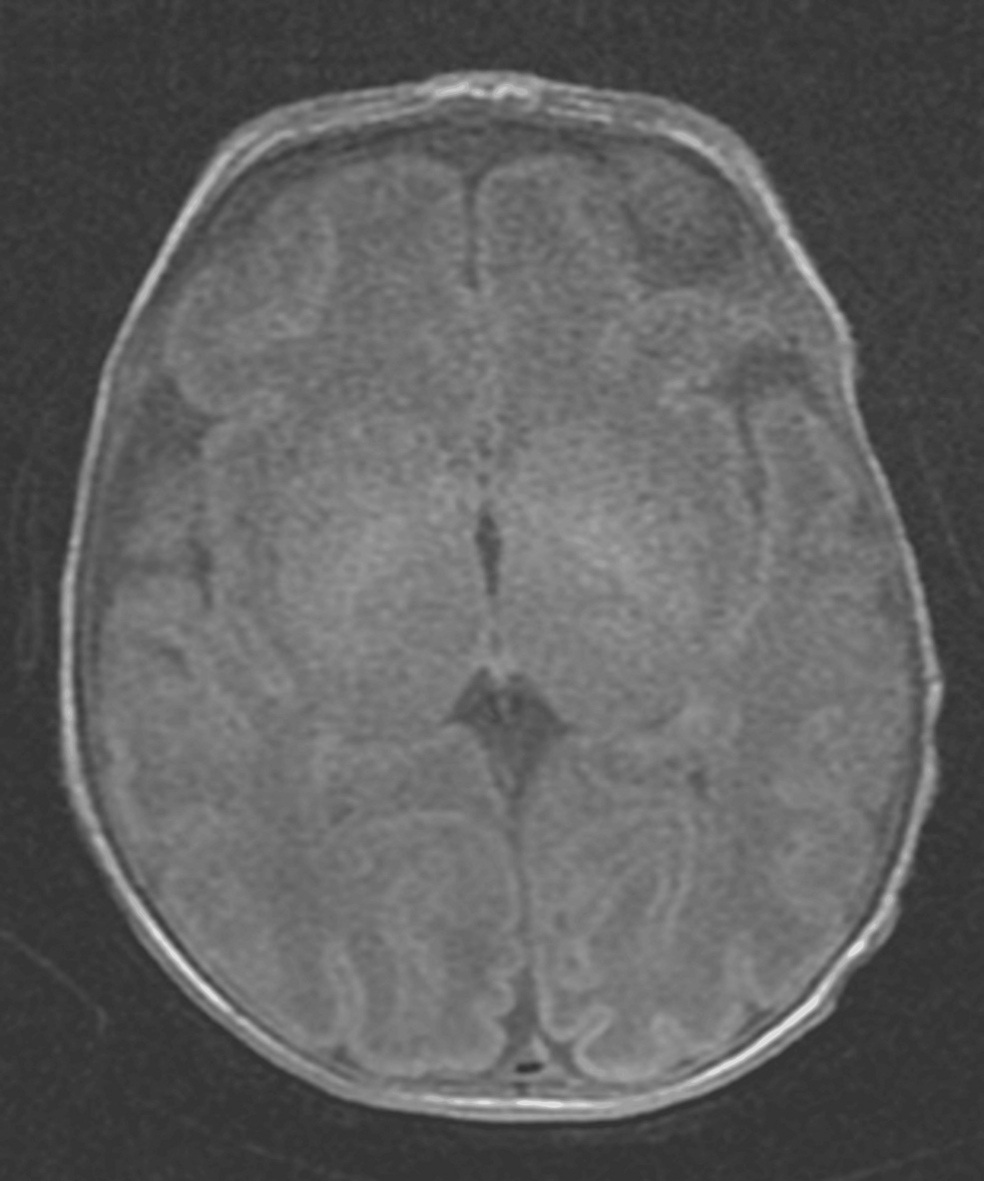
Normal brain MRI of our patient

An omphalocele covered by the unruptured translucent and avascular amniotic membrane measuring 10 cm. The rest of the malformation assessment noted ductus arteriosus with left superior vena cava, micro-polycystic kidneys, and bilateral pyelo-calyceal dilatation. The karyotype of the newborn revealed a trisomy 13. Brain MRI did not reveal other structural congenital malformations (Figure 4).

Ocular ultrasound reveals the absence of an eye globe and lens. The baby survived for seven days. He had surgery for his omphalocele. The post-operative consequences were complicated by the onset of cardiocirculatory failure and sepsis, the cause of his early death.

## Discussion

Trisomy 13 was first discovered in 1657 by Bartholin [4]. Later, in 1960, Patau defined its chromosomal anomaly [4]. The trisomy 13 incidence, which is the same in both sexes, is on the order of 1/10000 [5]. It may be released without fear of recurrence or as a result of a parental translocation. The risk of recurrence in this case is 5%. It has been established that a contributing factor to the occurrence of trisomy 13 is advanced maternal age. In this particular case, the mother was 40 years old, and age was considered a significant factor. There is no association between a higher prevalence of trisomy 13 and geographic location, race, or ethnicity [6].

Trisomy 13 has been associated with ophthalmic, genitourinary, cardiovascular, and severe central nervous system anomalies, as well as psychomotor retardation. Growth deficiency is a frequent condition that is linked to aneuploidy, poor feeding, gastrointestinal abnormalities, and gastro-oesophageal reflux, in addition to orofacial clefts. Moreover, cleft lips and palates exist in 80% of the cases. According to one series of cases, atrial septal defect, patent ductus arteriosus, and ventricular septal defect are the most frequent cardiac abnormalities, occurring in up to 80% of patients [7]. In more than 30% of cases, renal pathology, particularly cystic dysplasia, is documented. Up to 50% of patients have significant ocular pathology, including microphthalmia, colobomas, retinal dysplasia, and cataracts. Sensorineural hearing loss has also been documented [7].

Our patient exhibits bilateral anophthalmia, low-set ears, a median cleft lip and palate, a short neck, and polydactyly in the limbs, which are characteristic dysmorphic traits of trisomy 13.

Amniocentesis and chorionic villi samples can all be used to diagnose Patau syndrome during pregnancy. The characteristic deformities associated with Patau syndrome, including holoprosencephaly and other anomalies of the central nervous system, facial abnormalities, skeletal abnormalities, renal or cardiac problems, and growth limitation, can also be detected with the aid of prenatal ultrasound. The most sensitive time for prenatal ultrasonography to identify Patau syndrome anomalies is after 17 weeks of gestation. A cytogenetic analysis of fetal cells should be used to confirm any abnormal results [8].

Although Patau syndrome patients generally have a poor prognosis even after receiving intensive treatment. Because of their facial deformities, newborns with Patau syndrome may require a tracheostomy or intubation for post-delivery oxygenation and ventilation. Heart surgery may be necessary for patients with cardiac defects to correct common cardiac problems. For cleft lip repair, feeding tube placement, or corrective orthopedic procedures, additional surgeries can be necessary. The use of hearing aids, prophylactic antibiotics for urinary tract infections, customized nutritional feeds, and seizure prophylaxis are possible further treatments [8].

It is generally accepted that trisomy 13 has a poor prognosis. According to studies conducted by Magenis et al., 28% of the newborns who survived died in the first week, as in this case, 44% within a month and 86% during infancy [9].

## Conclusions

Trisomy 13 is an extremely rare autosomal trisomy that often leads to spontaneous abortion and death in the first few days or weeks of life. Because the related malformations are more severe and incompatible with life.

Patients with trisomy 13 usually have a poor life prognosis, which can be improved with prenatal diagnosis, multidisciplinary medical evaluation, and adequate genetic counseling.
